# 1-Methylcyclopropene counteracts ethylene promotion of fruit softening and roles of MiERF2/8 and *MiPG* in postharvest mangoes

**DOI:** 10.3389/fpls.2022.971050

**Published:** 2022-09-20

**Authors:** Rui Li, Jiheng Ma, Hui Gu, Wenjun Jia, Yuanzhi Shao, Wen Li

**Affiliations:** ^1^Key Laboratory for Quality Regulation of Tropical Horticultural Crops of Hainan Province, School of Horticulture, Hainan University, Haikou, China; ^2^Key Laboratory of Hainan Province for Postharvest Physiology and Technology of Tropical Horticultural Products, South Subtropical Crops Research Institute, Chinese Academy of Tropical Agricultural Sciences, Zhanjiang, China; ^3^School of Life Sciences, Hainan University, Haikou, China

**Keywords:** mango fruit, ethylene, softening, ripening, correlation analysis

## Abstract

Ethylene burst is an important sign of the initiation of postharvest mango ripening and softening is a typical characteristic of fruit ripening. However, the intrinsic link between ethylene release and fruit softening during ripening of postharvest mangoes is still not clear. The aim of this study was to investigate the effects of ethylene and its action inhibitor 1-methylcyclopropene (1-MCP) on fruit softening and ripening and the underlying regulatory mechanisms. Results showed that ethephon (ETH) promoted ethylene release and enhanced MDA content and activities of cell wall degrading enzymes, whereas 1-MCP treatment exhibited an opposite effect. Moreover, real-time quantitative polymerase chain reaction indicated that the transcription levels of genes involved in cell wall degradation (*MiPG*, *Miβ-GAL* and *MiPE*), ethylene biosynthesis (*MiACO1* and *MiACS6*) and ethylene response factor (*MiERF8*) were remarkably induced by ETH. Correlation analysis further revealed that the production of ethylene was significantly negatively correlated with firmness, but positively correlated with MDA content, activities of cell wall degrading enzymes and expressions of *MiPG* and *Miβ-GAL*. Furthermore, yeast one hybrid (Y1H) assay showed that MiERF2 and MiERF8 could directly bind to the promotor of *MiPG* and then regulate its transcription. These findings suggest that ethylene production is closely associated with fruit softening, and MiERF2 and MiERF8 and *MiPG* may play crucial roles in regulation of ripening and softening of postharvest mangoes.

## Introduction

Mango (*Mangifera indica* L.) is one of the most important fruit crops because of its unique flavor and rich nutritional value. However, the shelf life of postharvest mangoes is relatively short due to the burst of ethylene production and the climacteric rise of respiration rate (Zaharah et al., 2013). Therefore, improving fruit flavor and quality by effectively controlling ethylene is an important topic in postharvest preservation (Asif et al., 2022). Ethephon (2-chloroethylphosphonic acid, ETH) has been widely employed to hasten and homogenize fruit ripening (Tovar et al., 2011; Palafox-Carlos et al., 2012). 1-Methylcyclopropene (1-MCP), an ethylene action inhibitor, can reduce ethylene release and delay fruit softening and ripening, which contributes to the maintaining fruit quality and prolonging shelf life of many fruit species (Dias et al., 2021; Li et al., 2021).

Fruit softening is a complex metabolic process that results from major changes in the primary cell wall (Li et al., 2022b). Cell wall decomposition involves both enzymatic and non-enzymatic metabolic processes (Singh et al., 2019). Numerous cell wall modifying enzymes have been identified, such as polygalacturonase (PG), β-galactosidase (β-GAL), pectatelyase, pectin methylesterase (PE), cellulase (CX) and xylosidase. Among of them, PG, PE and CX are considered as the basic enzymes regulating the degradation of cell wall (Lin et al., 2020). Moreover, genes encoding pectin-degrading enzymes PG, β-GAL and PE are not only associated with softening of climacteric fruit (Cao et al., 2014), but only closely related with softening of non-climacteric fruit (Sun et al., 2022). However, the key genes responsible for softening are obviously different in different fruit species and varieties. For example, *PcPG1* and *PcPG2* are significantly increased in ‘France’ pears, whereas they are not expressed in ‘Yali’ pears during ripening (Zhai et al., 2018).

Ethylene release and fruit softening are important characteristics in ripening process (Razzaq et al., 2013). Ethylene release is closely related with the changes in activities of pectin-degrading enzymes that contributes to the softening of postharvest apples (Gwanpua et al., 2017). Moreover, ethylene is necessary for triggering PG synthesis and the increased production of ethylene occurs prior to the production of PG (Gaete-Eastman et al., 2009). Many regulatory sequences associated to ripening have been identified in *PGs* promoter regions, which supports the important roles of *PGs* in softening during mango fruit ripening (Dautt-Castro et al., 2019). Furthermore, ethylene responsive factor (ERF) is a subfamily of plant AP2/ERF transcription factor superfamily and widely involved in regulation of plant growth, development, and fruit ripening (Gao et al., 2020). During ripening and softening of persimmon fruit, the transcription levels of *ERF22* and *PG1* are both significantly up-regulated (Kou et al., 2021).

Mango is a typical respiratory climacteric fruit and its general softening and ethylene behavior have been well documented (Tovar et al., 2011; Zaharah et al., 2013; Chen et al., 2022). During ‘Samar Bahisht Chaunsa’ mango fruit ripening, activities of exo-PG, endo-PG and endo-1,4-β-d-glucanase gradually increase and activity of PE decreases, which coordinately controls mango fruit softening (Razzaq et al., 2013, 2014, 2015). Moreover, the genes responsible for ethylene biosynthesis (*ACS*, *ACO*) and cell wall metabolism (*PG14*, *PG21*, *PEL*) play key roles in different ripening stages of ‘Keitt’ mango (Busatto et al., 2022). However, the relationship between ethylene production and fruit softening during postharvest mango ripening is still not clear.

In this study, fruit softening and ethylene behaviors under different ripening control methods were investigated during storage of ‘Hongyu’ mango at 15°C. Ethylene production, respiratory rate, malondialdehyde (MDA) content, activities of cell wall degradation enzymes including PG, β-GAL and CX, and expressions of corresponding enzyme genes were detected after treatment. Moreover, correlation analysis among the above indicators were carried out to study the intrinsic link between ripening and softening. Yeast one hybrid (Y1H) assay was employed to explore the possible regulatory mechanism mediated by ethylene signals on fruit softening.

## Materials and methods

### Fruit materials

All mango (*M. indica* L. cv. Hongyu) fruit were harvested at mature green stage (about 120 days after full flowering) from a commercial orchard located in Changjiang city, Hainan province, China. The mangoes were packed in plastic crates, wherein the fruit of each layer were separated with soft fabric, and transported to the postharvest laboratory (25°C, 75–80% relative humidity) of Hainan University within 4 h. Only well-formed fruit that were free of disease and blemishes and with uniformed color, shape and size were selected out for this study. The selected fruit were soaked with 0.1% sodium hypochlorite (Zhuhai Huimay Biotech Co. Ltd., Gold Yocht®) for 10 min, and then immersed in 0.1% Sporgon (Sporgon, FMC Crop., United States) for 10 min, air-dried at 25°C, and used for further experiments.

### Postharvest treatment

All mango fruit were divided randomly into three groups, and each group was consisted of 100 mangoes. The first group was soaked in 0.8 g L^−1^ ethephon (ETH, Huanyuan Chemical Co., Ltd., Shanghai, China) solution for 5 min and closed in a plastic bag for 24 h at 25°C. The second group was fumigated with 1 μl L^−1^ 1-MCP (Hepeng Biotechnology Co., Ltd., China) for 20 h in a closed foam box at 25°C. The third group has no treatment and was used as the control. Subsequently, all fruit were placed in 0.02 mm thick PE bags (Jieyang Gang Tai^®^, China) and stored at 15 ± 0.5°C temperature with 85–90% relative humidity for up to 30 days.

For determination of fruit firmness, texture indicators, ethylene production and respiration rate, the intact fruit were picked every 5 days. Afterwards, the flesh samples of these fruit were collected and rapidly frozen in liquid nitrogen, and then kept at −80°C for analysis of other indicators. At each sampling time point, all the treatments were conducted using three biological replicates of nine mango fruit (3 fruit for 1 replicate).

### Determination of ripening-related parameters

#### Ethylene production

The production of ethylene was determined following the procedures described by Chen et al. with minor modifications (Chen et al., 2022). Three mango fruit were weighed and sealed in a 12 l airtight jar for 2 h at 25°C. Three 1 ml gas samples were collected from each vessel by syringe and injected into a gas chromatograph (Agilent 5,181–1,267, Palo Alto, CA, United States) equipped with a flame ionization detector (Thermo Fisher Scientific, TRACE 1300, United States). The peaks were compared to the standard gas sample of ethylene. Ethylene production was calculated and expressed as μL g^−1^ h^−1^.

#### Respiration rate

Three mango fruit were weighed and sealed in a 12 l airtight jar for 2 h at 25°C. 1 ml of gas was extracted for measuring contents of O_2_ and CO_2_ with a portable O_2_/CO_2_ headspace analyzer (Dansensor^®^ CheckPoint3, Dan Saint, Denmark). The respiration rate was calculated and expressed in (CO_2_) mL kg^−1^ h^−1^.

#### MDA content

MDA content was determined according to our previous reports with minor modifications (Shao et al., 2012; Chen et al., 2022). The 6 g of fresh pulp were mixed with 6 ml of 0.05 M (pH = 7.8) phosphate buffer and centrifuged at 13,500 rpm for 30 min. The 2 ml of supernatant was added to 3 ml of 10% TCA with 0.5% thiobarbituric acid, rapidly boiling water bath for 10 min, and then centrifuged at 13,500 rpm for 30 min. The absorbances of supernatant at 532 nm and 600 nm were used for MDA content. The results were presented as nmol g^−1^.

### Determination of softening-related parameters

#### Fruit firmness, chewiness, adhesiveness, springiness, and cohesiveness

One TA touch texture profile analyzer (Bosin Tech, Shanghai, China) equipped with a 2 mm diameter probe was used to measure fruit firmness, chewiness, adhesiveness, springiness, and cohesiveness. It was inserted into fruit pulp without peel at the angle of 90° and depth of 5 mm at two equatorial sites. The operational parameters were as follows: pre-test speed, 2 mm s^−1^; test speed, 4 mm s^−1^; post-test speed, 3 mm s^−1^, and intermediate interval, 2 s.

#### Contents of protopectin and soluble pectin

Protopectin and soluble pectin were extracted based on the protocol reported by Zhao et al. (2019). Briefly, the 1 g frozen pulp was ground in 25 ml 95% ethanol, The mixture was incubated on 100°C water bath for 30 min, cooled to room temperature, and this step was repeated three times to remove sugar and other substances from samples. Afterwards, the mixture was filtered and the filter residues were washed twice with 75% ethanol and then dried at room temperature.

For soluble pectin content, the alcohol insoluble solids were suspended in 20 ml sterile water, incubated at 50°C for 30 min and centrifuged at 8,000 rpm for 15 min to obtain soluble pectin. For protopectin content, the alcohol insoluble solids were suspended in 0.5 mol L^−1^ sulfuric acid solution and incubated on 100°C water bath for 1 h to hydrolyze protopectin. Finally, the contents of soluble pectin and protopectin were measured according to the carbazole colorimetry method employing galacturonic acid as a standard (Zhang et al., 2008).

#### Activities of PG, CX and β-GAL

PG and CX activities were measured according to the protocol of (Wang et al., 2018). The 2 g of frozen pulp was homogenized with 8 ml of pre-cooling 95% ethanol. The mixture was transferred to a 10 ml centrifuge tube and stood for 10 min at 4°C. Subsequently, 3 ml of 2 mol L^−1^ H_2_SO_4_ was added into the tube, and then centrifuged at 13,500 rpm for 10 min. The residue was dissolved with 5 ml of pre-cooled 50 mmol L^−1^ (pH = 5.5) sodium acetate buffer and centrifuged at 13,500 rpm for 10 min at 4°C.

For PG activity, the assay system contained 0.5 ml of 0.1% polygalacturonic acid (PGA), 1 ml of 0.1 mol L^−1^ sodium acetate buffer (pH = 4.6), 0.5 ml distilled water and 1 ml enzyme extract. The mixture was incubated at 37°C for 1 h, and the reaction was terminated with 1 ml DNS (3,5-dinitrosalicylic acid). The absorbance at 540 nm was analyzed. d-Galacturonic acid was employed as a standard and PG activity was expressed as mg g^−1^ h^−1^.

For CX activity, the reaction mixture consisted of 1.5 ml of 10 g L^−1^ sodium carboxymethyl cellulose solution, 1 ml of 0.2 mol L^−1^ sodium acetate buffer (pH = 4.6), and 0.5 ml enzyme extract, and the mixture was incubated at 37°C for 1 h. The reaction was terminated with 1.5 ml DNS, and then the mixture was placed into boiling water for 5 min and cooled to room temperature. The absorbance at 540 nm was analyzed. Glucose was employed as a standard and CX activity was expressed as mg g^−1^ h^−1^.

For β-GAL activity, 1 g of fruit pulp was homogenized with 8 ml of 100 mmol L^−1^ citric acid buffer (pH = 5.0), centrifuged at 11,500 rpm and 4°C for 30 min, and the supernatant was used for determination of β-GAL activity (Wei et al., 2010). The assay system contained 1 ml of 20 mmol L^−1^ o-nitrophenyl-β-d-galactopyranoside solution, 1 ml of 100 mmol L^−1^ citric acid buffer (pH = 5.0) and 1 ml of enzyme extract. The mixture was incubated at 40°C for 20 min. Afterwards, 1.5 ml of pre-cooled 500 mmol L^−1^ Na_2_CO_3_ solution and 2.5 ml distilled water were added in mixed solution. The absorbance at 420 nm was analyzed. Galactose was employed as a standard and β-GAL activity was expressed as mg g^−1^ h^−1^.

### Measurement of gene expression

#### Total RNA extraction and cDNA synthesis

The extraction of total RNA was carried out with cetyltrimethylammonium bromide (CTAB) method with slight modifications (Ma et al., 2018). The A260/A280 ratio and agarose gel electrophoresis were used to verify RNA integrity and quality. One PrimeScript™ RT reagent Kit with gDNA Eraser (HiScript^®^, Nanjing, China) was used for cDNA synthesis according to the manufacturer’s instructions.

#### Real-time quantitative polymerase chain reaction (RT-qPCR)

The SYBR Premix Ex Taq (HiScript^®^, Nanjing, China) was used for RT-qPCR following the manufacturer’s instruction. The primers were designed with Primer explorer v5 online website^1^ and were listed in Supplementary Table S1. The thermal cycling protocol comprised an initial denaturation at 95°C for 5 min, followed by 40 cycles of 95°C for 5 s and 60°C for 30 s. The RT-qPCR was amplified with a qTOWER3 G Real-Time PCR System (Wacker Biotech GmbH, Germany). *MiActin* was used as the reference gene and the relative expressions were determined using the 2^−ΔΔCt^ method.

### *Cis*-Element analysis of *MiPG* promoter

The sequence of *MiPG* promoter was obtained from a genome database (https://www.ncbi.nlm.nih.gov/). Total genomic DNA was extracted using a Plant Genomic DNA Kit (TIANGEN, Beijing, China). Analysis of *cis*-elements in the promoter region of *MiPG* was conducted with PlantCARE online database.^2^ Primer pair used for cloning of *MiPG* promoter were listed in Supplementary Table S1.

### Yeast one hybrid assay

Y1H assay was carried out using the Matchmaker Gold Y1H System (Clontech, CA, United States). *MiERF2* and *MiERF8* were, respectively, cloned into pGADT7 to generate the pGADT7-*MiERF2*/*8*. The 2076 bp of *MiPG* promoter was cloned into pHIS2 vector to generate pHIS2-*MiPG*. The primers used for recombinant plasmids were listed in Supplementary Table S1. Yeast strain Y187 co-transformed with pGADT7-*MiERF2* (or pGADT7-*MiERF8*) and pHIS2-*MiPG* were cultivated on SD/-Trp/-His for 3 days, and then positive colonies were inoculated on SD/-Trp/-His/-Leu/100 mM 3-AT. Y187 yeast strain containing pHIS2-53 and pGADT7-53 were used as positive control, and Y187 strain containing pHIS2 and pGADT7-53 were used as negative control.

### Statistical analysis

The data are presented as means ± standard deviations. Duncan’s multiple range tests were used to verify the significance differences at *p* = 0.05 with SPSS software 16.0 (SPSS Inc., Chicago, IL, United States). Correlation analysis was carried out *via* OriginPro 2021 (Origin Lab, United States) with differences being considered significant (^*^p < 0.05, ^**^p < 0.01, ^***^p < 0.001).

## Results

### Effects of different treatments on ripening of mango fruit during cold storage

**Figure 1:**
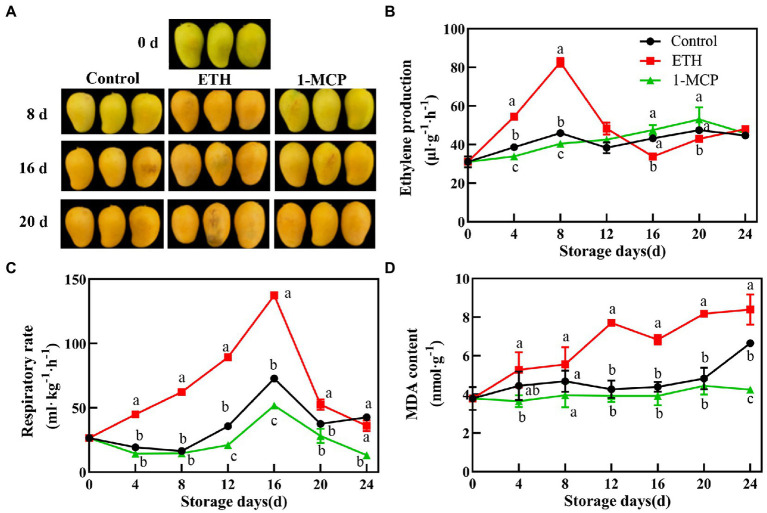
Effects of different treatments on ripening phenotype **(A)**, ethylene production **(B)**, respiratory rate **(C)** and MDA content **(D)** in mango fruit during storage at 15°C. The data are presented as means ± standard deviations. Means with different letters are significantly different at *p* < 0.05.

In the present study, ETH treatment accelerated the ripening process of postharvest mangoes, whereas 1-MCP treatment showed an opposite effect (Figure 1A). ETH treatment enhanced the production of ethylene, which reached the peak value on 8 days with 82.67 μl h^−1^ g^−1^. The peak of ethylene production in control fruit also occurred on 8 days, but it was significantly lower than that in ETH treated fruit. However, 1-MCP treatment delayed the production of ethylene, which peaked on 20 days (Figure 1B, *p* < 0.05). Moreover, respiration rate in the three groups exhibited a similar change trend during the whole storage time and they all peaked on 16 days. As shown in Figure 1C, ETH treatment induced the highest respiration rate of mango fruit, followed by control group, and the last was 1-MCP treated group (*p* < 0.05). Compared with control, MDA content of ETH treated fruit significantly increased, while 1-MCP treatment significantly suppressed the increase in MDA content until 24 days (Figure 1D, *p* < 0.05).

### Effects of different treatments on fruit texture properties during cold storage

As shown in Table 1, control, ETH and 1-MCP treated fruits exhibited significant differences in texture indicators from the eighth day (*p* < 0.05). Firmness of three group fruits all decreased with the extension of the storage time. Compared with control, ETH treatment remarkably accelerated the decrease in firmness, while 1-MCP significantly inhibited the decrease. For example, the firmness of ETH or 1-MCP treated fruit was 66.3% lower or 48.86% higher than that of control on day 24. Moreover, chewiness and adhesiveness of ETH treated fruit were significantly lower than those of control on 8, 16 and 24 days (*p* < 0.05). However, adhesiveness of 1-MCP treated fruit was significantly higher than that of control on 16 and 24 days (*p* < 0.05). Springiness in the three groups showed no significantly difference on 0, 8 and 16 days (*p* < 0.05), but ETH treated fruit exhibited a significantly lower springiness than control and 1-MCP treated fruits on 24 days. Additionally, the values of cohesiveness in 1-MCP treated fruit showed no significantly difference during the whole storage time.

**Table 1:** Changes of flesh firmness, chewiness, adhesiveness, springiness, and cohesiveness during storage at 15°C.

Storage time (*d*)	Treatment	Firmness (*n*)	Chewiness (*n*)	Adhesiveness (*n*)	Springiness (*n*)	Cohesiveness (*n*)
0	Control	32.2 ± 1.36a	13.5 ± 0.85a	12.3 ± 0.65a	1.15 ± 0.05a	0.40 ± 0.02a
	ETH	32.2 ± 1.36a	13.5 ± 0.85a	12.3 ± 0.65a	1.15 ± 0.05a	0.40 ± 0.02a
	1-MCP	32.2 ± 1.36a	13.5 ± 0.85a	12.3 ± 0.65a	1.15 ± 0.05a	0.40 ± 0.02a
8	Control	27.8 ± 1.15a	11.0 ± 0.12a	9.6 ± 0.36a	0.98 ± 0.04a	0.35 ± 0.02a
	ETH	12.5 ± 0.88b,c	4.5 ± 0.08b,c	3.1 ± 0.02b,c	0.95 ± 0.06a	0.28 ± 0.01b
	1-MCP	29.8 ± 1.25a	11.5 ± 0.14a	12.2 ± 0.56a	1.10 ± 0.05a	0.38 ± 0.03a
16	Control	23.5 ± 1.38a	8.5 ± 0.14a	7.5 ± 0.46b	0.94 ± 0.0.07a	0.31 ± 0.01a
	ETH	7.85 ± 0.07b,c	1.86 ± 0.07b,c	1.8 ± 0.08b,c	0.91 ± 0.03a	0.20 ± 0.01b
	1-MCP	27.8 ± 1.54a	9.5 ± 0.14a	11.8 ± 0.88a	0.98 ± 0.06a	0.36 ± 0.02a
24	Control	13.5 ± 0.18b	7.2 ± 0.12a	7.7 ± 0.66b	0.85 ± 0.02a	0.25 ± 0.02b
	ETH	4.55 ± 0.05b,c	0.65 ± 0.07b,c	0.6 ± 0.04b,c	0.78 ± 0.04b	0.12 ± 0.02b,c
	1-MCP	26.4 ± 1.82a	7.83 ± 0.58a	10.5 ± 0.63a	0.94 ± 0.05a	0.38 ± 0.02a

Different letters indicate statistically significant differences (*p* < 0.05).

### Effects of different treatments on contents of main structure component of cell wall

**Figure 2:**
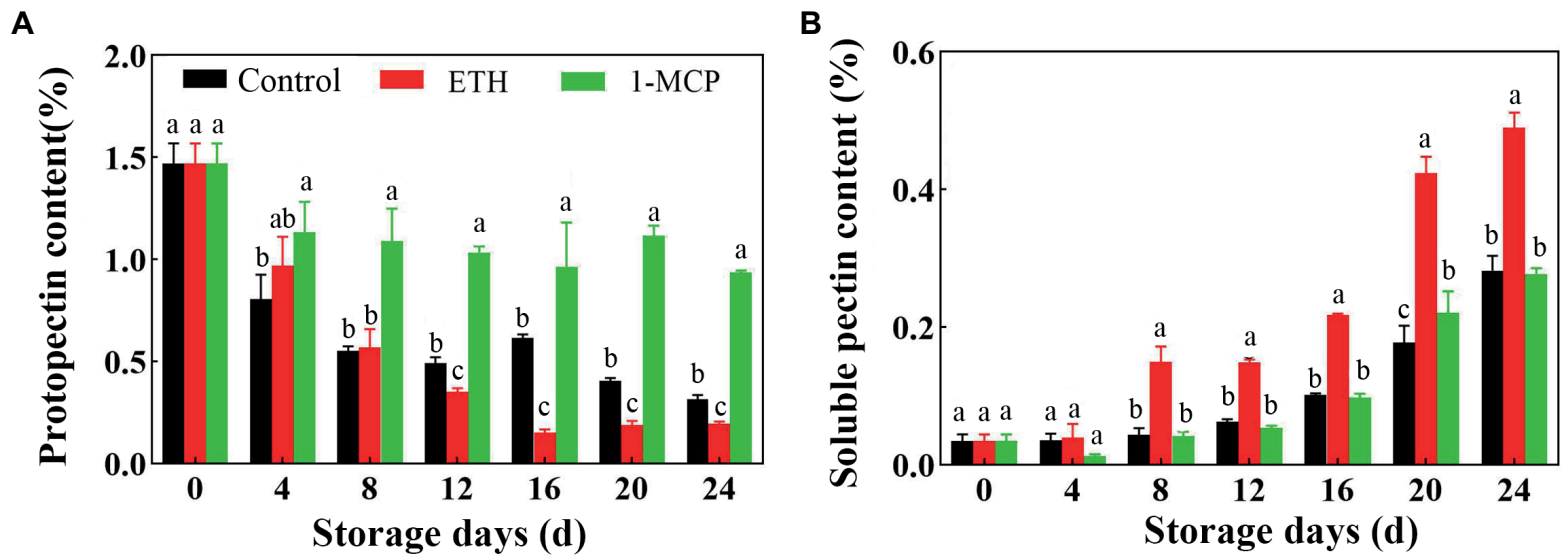
Effects of different treatments on contents of protopectin **(A)** and soluble pectin **(B)** in mango fruit during storage at 15°C. The data are presented as means ± standard deviations. Means with different letters are significantly different at *p* < 0.05.

With the prolongation of storage time, the content of protopectin in the three groups generally showed a downward trend (Figure 2A). Compared with control, ETH treatment decreased the contents of protopectin on 12, 16, 20 and 24 days. However, 1-MCP treated fruit showed significantly higher contents of protopectin than control during the whole storage time (Figure 2A, *p* < 0.05). The content of protopectin in 1-MCP treated fruit was 49.2% and 47.8% higher than that in control and ETH treated fruits on day 8, respectively (Figure 2A). Additionally, the content of soluble pectin all exhibited an upward trend in the three group fruits, and ETH treated fruit showed significantly higher content of soluble pectin than control and 1-MCP treated fruits (Figure 2B, *p* < 0.05). However, there was no significant difference on the content of soluble pectin between control and 1-MCP treated fruits (Figure 2B).

### Effects of different treatments on activities of enzymes involved in cell wall metabolism

**Figure 3:**
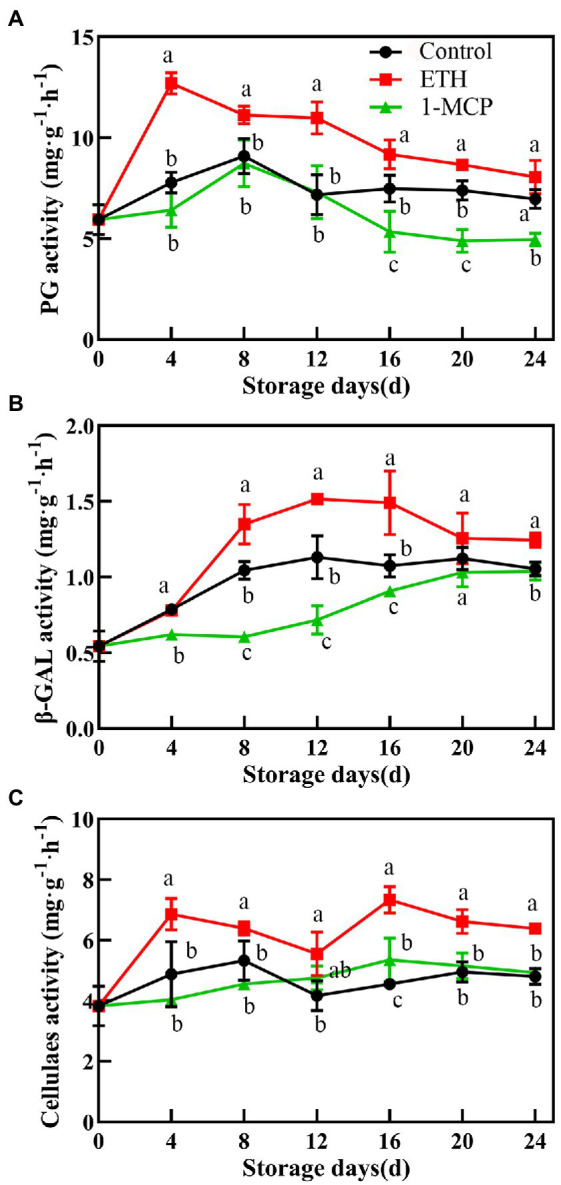
Effects of different treatments on activities of polygalacturonase **(A)**, β-galactosidase **(B)**, and cellulase **(C)** in mango fruit during storage at 15°C. The data are presented as means ± standard deviations. Means with different letters are significantly different at *p* < 0.05.

ETH treatment significantly increased the PG activity, which peaked on day 4, and the value was 38.7% and 49.4% higher than those of control and 1-MCP treated fruits, respectively (Figure 3A, *p* < 0.05). Compared with control, 1-MCP treatment effectively reduced the activities of PG and β-GAL during the whole storage time (Figures 3A,B). The β-GAL activity in 1-MCP treated fruit was significantly lower than those in control and ETH treated fruits through all storage period (Figure 3B, *p* < 0.01). The activity of CX in 1-MCP treated and control fruits exhibited no significantly difference (Figure 3C). However, the peak activities of CX in ETH treated fruit were significantly higher than that in control and 1-MCP treated fruits on day 14 (Figure 3C, *p* < 0.05).

### Effects of different treatments on expression of genes involved in fruit softening and ripening

**Figure 4:**
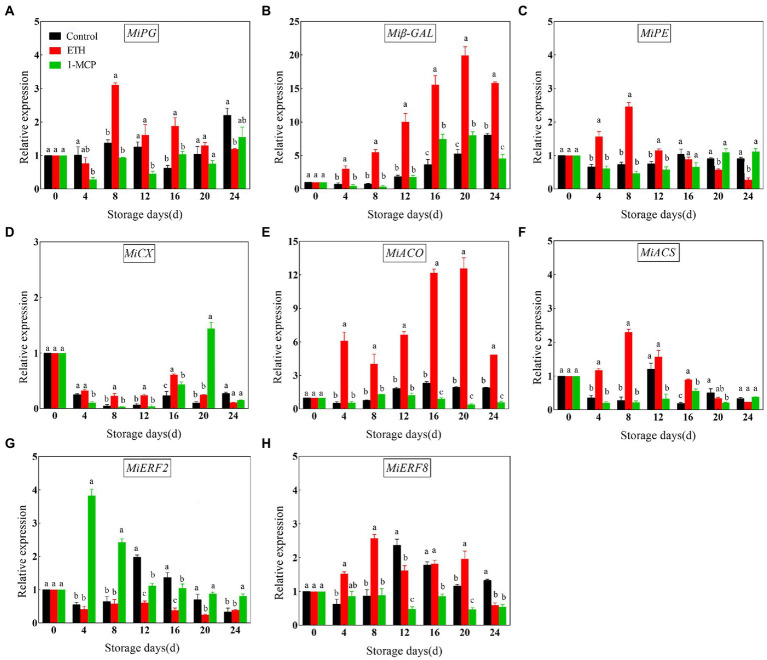
Effects of different treatments on expression levels of *MiPG* **(A)**, *Miβ-GAL* **(B)**, *MiPE* **(C)**, *MiCX* **(D)**, *MiACO1* **(E)**, *MiACS6* **(F)**, *MiERF2* **(G)** and *MiERF8* **(H)** in mango fruit during storage at 15°C. The data are presented as means ± standard deviations. Means with different letters are significantly different at *p* < 0.05.

To further investigate the correlation between fruit softening and ripening, the transcription levels of genes related with ethylene synthesis and cell wall degradation were measured *via* RT-qPCR. Compared with control, ETH treatment significantly up-regulated the expression levels of *MiPG*, *Miβ-GAL*, *MiPE*, *MiACO1*, *MiACS6* and *MiERF8* and down-regulated the expression levels of *MiERF2* (Figure 4, *p* < 0.05). However, 1-MCP treatment showed a converse effect, and it significantly inhibited the transcription of *MiPG*, *Miβ-GAL*, *MiPE*, *MiACO1*, *MiACS6* and *MiERF8* and promoted the transcription of *MiERF2* (Figure 4, *p* < 0.05). In addition, the results showed that the promoting effect of ETH and the suppressing effect of 1-MCP on *MiCX* transcription were not more pronounced than the other genes (Figure 4D, *p* < 0.05).

### Correlation analysis between parameters involved in fruit ripening and softening

**Figure 5:**
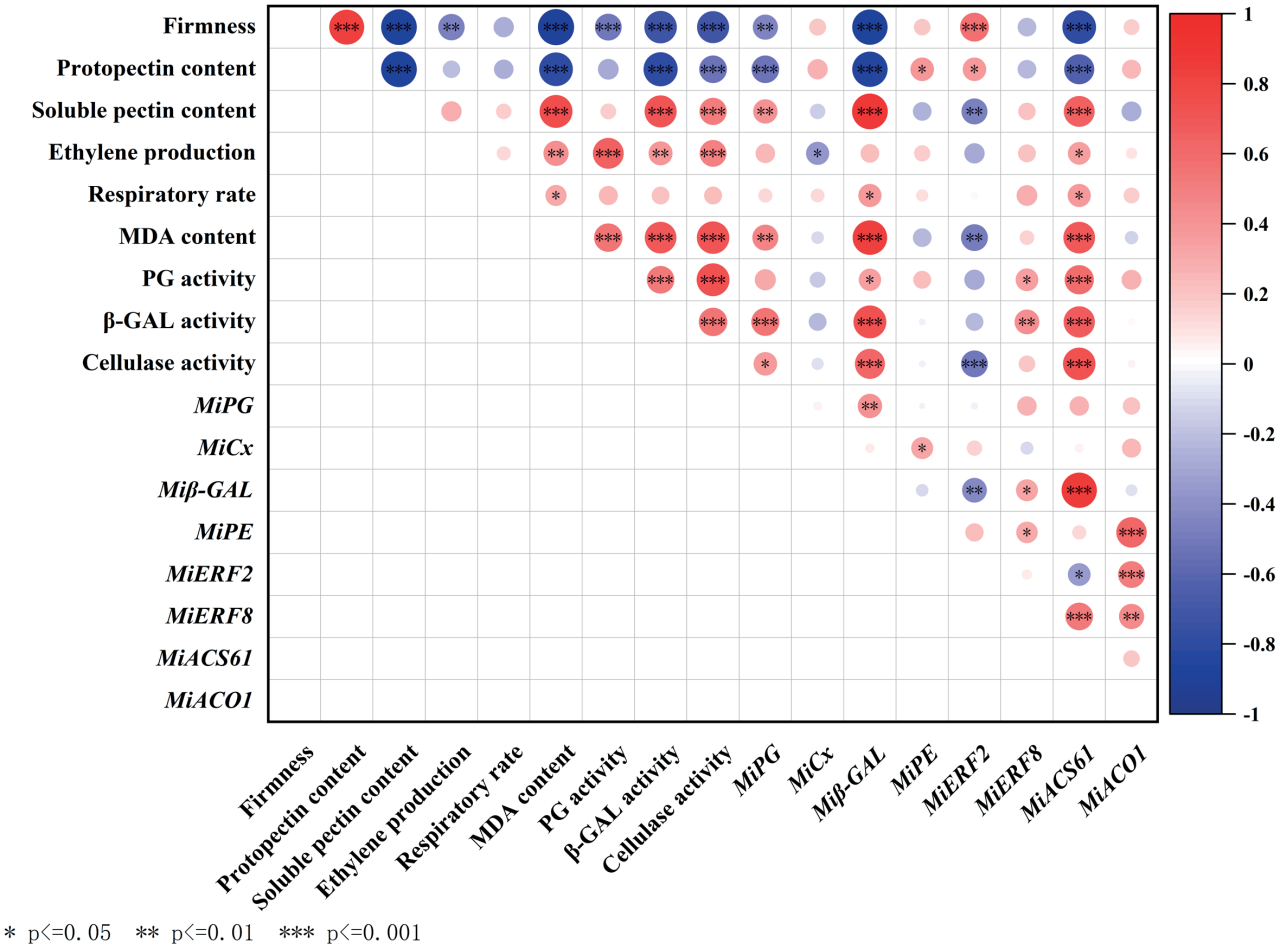
Correlation analysis between parameters including firmness, protopectin content, soluble pectin content, ethylene production, respiratory rate, MDA content, activities of PG, *β*-GAL and cellulase, and expressions of *MiPG*, *Miβ-GAL*, *MiCX*, *MiPE*, *MiERF2*, *MiERF8*, *MiACO1* and *MiACS6*. Asterisks indicate significant differences as determined by Duncan’s multiple range test (^*^p < 0.05, ^**^p < 0.01 and ^***^p < 0.001).

Firstly, we analyzed the correlations among fruit ripening indicators (Figure 5). It can be found that MDA content was significantly positively correlated with ethylene production and respiratory rate, and the Pearson’s correlation coefficients (R value, as follows) were 0.43 (*p* < 0.01) and 0.31 (*p* < 0.05), respectively. Moreover, *MiACS6* but not *MiACO1* significantly positively correlated with ethylene production (*R* = 0.35, *p* < 0.05), respiratory rate (*R* = 0.37, *p* < 0.05), and MDA content (*R* = 0.7, *p* < 0.001). *MiERF2* but not *MiERF8* significantly negatively correlated with MDA content (*R* = −0.49, *p* < 0.01).

Furthermore, the correlations among fruit softening indicators were analyzed. Results showed that firmness was significantly positively correlated with protopectin content and negatively correlated with soluble pectin content, the *R* value were 0.83 and −0.88, respectively (*p* < 0.001). Protopectin content negatively correlated with soluble pectin content (*R* = −0.88, *p* < 0.001), activities of β-GAL (*R* = −0.79, *p* < 0.001) and CX (*R* = −0.53, *p* < 0.001), and expression of *MiPG* (*R* = −0.52, *p* < 0.001) and *Miβ-GAL* (*R* = −0.85, *p* < 0.001), but significantly positively correlated with *MiPE* expression (*p* < 0.05). However, firmness exhibited no significant correlation with expression of *MiCX* or *MiPE* (*p* < 0.05). In addition, the significantly negatively correlations between firmness and activities of PG, β-GAL, and CX were also observed.

To study the correlation between fruit ripening and softening, R values between the above ripening and softening indicators were further analyzed (Figure 5). Results showed that ethylene production was negatively correlated with firmness (*R* = −0.48, *p* < 0.01). Moreover, MDA content significantly negatively correlated with firmness (*R* = −0.90, *p* < 0.001) and protopectin content (*R* = −0.78, *p* < 0.001), but positively correlated with soluble pectin content (*R* = 0.75, *p* < 0.001). Both ethylene production and MDA content significantly positively correlated with activities of PG, β-GAL and CX (*p* < 0.01). Furthermore, *MiERF2* but not *MiERF8* significantly positively correlated with fruit firmness and protopectin content (*p* < 0.05).

### Interaction between MiERF2 or MiERF8 and *MiPG*

**Figure 6:**
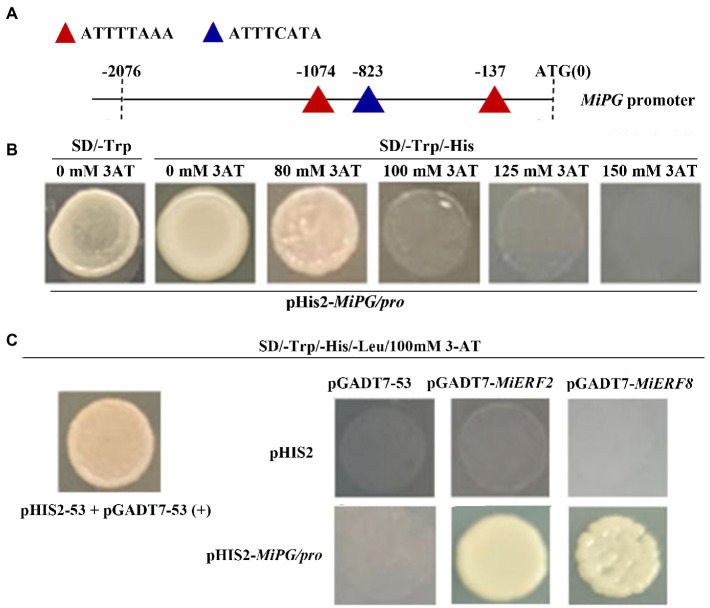
Analysis of the interaction between MiERF2 (or MiERF8) and *MiPG* promoter. **(A)** *Cis*-acting element analysis of *MiPG* promoter. **(B)** Screening of 3AT concentrations that prevent the auto-activation of *MiPG* promoter. **(C)** The interaction between MiERF2 (or MiERF8) and *MiPG* promoter *via* yeast one-hybrid.

To further investigate the positive correlation between ethylene production and fruit softening, the interaction between MiERF2 (or MiERF8) and *MiPG* was analyzed *via* Y1H. As shown in Figure 6A, three ethylene responsive element (ERE) were found in *MiPG* promoter region. Moreover, yeast strain transformed with pHIS2-*MiPG* promoter grew well on SD/-Trp/-His/80 mM 3-AT, but its growth was completely inhibited on SD/-Trp/-His/100 mM 3-AT (Figure 6B), which indicates that 100 mM 3-AT was appropriate for the following experiments. Furthermore, similar with positive control and contrary to negative control, yeast strain co-transformed with pHIS2-*MiPG* promoter and pGADT7-*MiERF2* or pGADT7-*MiERF8* exhibited normal growth on SD/-Trp/-His/-Leu/3-AT (Figure 6C).

## Discussion

Ethylene is a necessary regulator in the initiation and progression of fruit softening (Li et al., 2022b). Ethylene release and respiratory rate are closely related to softening and quality deterioration of postharvest fruit (Silué et al., 2022). It is consistent with our results that 1-MCP treatment decreased MDA content and respiratory rate and maintained the firmness of mango fruit (Figure 1). Furthermore, the positive correlation between ethylene production and MDA content was found (Figure 5). However, fruit firmness was significantly negatively correlated with ethylene production and activities of PG, β-GAL, CX and expressions of *MiPG* and *Miβ-GAL* (Figure 4). These results suggest that ethylene signal pathway is closely related to fruit softening.

Texture is one of the important commodity features that determine postharvest fruit quality and consumer acceptability. Firmness is also an important indicator that reflects fruit texture characteristics. Fruit softening is always accompanied by loss of firmness, which further lead to quality deterioration of postharvest fruit (Ren et al., 2020). In this study, mango fruit softening is accompanied not only by the decrease in pulp firmness, but also declines in adhesiveness, springiness, cohesiveness, and chewiness (Table 1). This result is supported by the results in Zaharah and Singh (2011). Moreover, fruit firmness can be affected by different postharvest treatments. For example, 1-MCP treatment could significantly suppress fruit softening and maintain texture characteristics, whereas ETH treatment may accelerate softening and reduce firmness during storage of mango fruit. Similar results have also been reported in plum (Lin et al., 2018) and persimmon (Wang et al., 2020) fruit.

Previous studies have shown that fruit softening is a consequence of modifications of the pectin in middle lamellae and the polymers in primary cell wall (Bu et al., 2013). In this work, contents of protopectin decreased and soluble pectin increased during storage of mango fruit at 15°C (Figures 2A,B). Moreover, protopectin levels of 1-MCP treated mangoes were higher than those of control and ETH treated fruits, but soluble pectin contents of ETH treated fruit were lower than those of control and 1-MCP treated mangoes, indicating that ETH treatment could accelerate degradation of protopectin and accumulation of soluble pectin in postharvest mango fruit, and then lead to fruit softening. Similar results have been reported in Japanese plum (Khan and Singh 2009) and fig (Ren et al., 2022) fruit.

Moreover, fruit firmness has been confirmed to be associated with the suppressed activities of cell wall modifying enzymes (Chea et al., 2019). Ripening induced the degradation of pectin and cellulose, and the contents of water-soluble pectin gradually increased from the lowest to highest ripening stage (Li et al., 2022a). To clarify fruit softening process mediated by ETH and 1-MCP, activities of enzymes responsible for degradation of cell wall were further investigated. Results showed that ETH treatment accelerated the degradation of protopectin, which was accompanied by the enhanced activities of PG, β-GAL and CX (Figures 2A, 3). 1-MCP treatment suppressed activities of PG, β-GAL and CX and played a positive role on maintenance of higher fruit firmness (Figure 3; Table 1), which was supported by the results in Win et al. (2021). Similar results in ‘Guifei’ mango have shown that application of melatonin suppressed the changes in activities of PG, β-GAL, and PE, which contributes to delaying fruit ripening and softening (Liu et al., 2020). Furthermore, among the three enzymes, activities of PG increased quickly and reached the peak value on 4 days, followed by β-GAL (Figures 3A,B).

Cellulase is one of imperative enzymes correlated with fruit softening, and the enhanced activity of CX leads to pectin solubilization and degradation in fruit cell wall (Ge et al., 2020). Carboxymethyl cellulose coating can delay the ripening process *via* regulating activities of softening enzymes in harvested mangoes (Ali et al., 2022). In blueberry (Wang et al., 2018) and apple (Lu et al., 2018) fruit, CX contributes to remarkably reducing the softening rapid. However, in this study, compared with ETH treated fruit, CX activities in control and 1-MCP treated fruits were significantly lower during the whole storage time, but there were not obvious differences between the two groups (Figure 3C). Additionally, the correlation analysis showed that the expression of *MiCX* did not significantly correlate with firmness (Figures 4D, 5). These findings suggest that fruit softening is complex process (Chen et al., 2021) and the involved enzymes responsible for cell wall degradation vary among different fruits.

Recently, the regulatory mechanisms of fruit softening have attracted more attentions. Nakatsuka et al., (2011) indicated that fruit softening was related to both *DkXTH1* and *DkXTH2* in ‘Saijo’ persimmons. *FaExpA2* has a high accumulation rate during fruit ripening of four strawberry cultivars (Valenzuela-Riffo and Morales-Quintana, 2020). In ‘Zill’ mango fruit, expression of *MiExpA1* in peel and the flesh contributes to alleviating cell wall degradation (Zheng et al., 2012). In the present study, the expressions of *MiPG* and *Miβ-GAL* in ETH treated fruit displayed similar continuous increasing trends with the extension of storage time (Figures 4A,B), which were consistent with the changes in activities of PG and β-GAL (Figures 3A,B). Moreover, firmness significantly negatively correlated with expression of *MiPG* and *Miβ-GAL* (Figure 5). These results indicate that *MiPG* and *Miβ-GAL* might play important roles in fruit softening during ETH induced fruit ripening process.

Furthermore, ERFs are the key transcription factors responsible for ethylene downstream signal transduction. Previous study in peach fruit reported that PpERF4 enhances the transcription of *PpACO1* by binding to its promoter in (Wang et al., 2021). ERF4 also represses the expression of *ACS1* and *ACO1* by interacting with JAZ in apple fruit (Hu et al., 2022). Similar results were reported in blueberry (Zhou et al., 2021). In this study, *MiERF2 and MiERF8* all exhibited significant correlations with *MiACS6* and *MiACO1* (Figure 5), indicating that *MiERF2* and *MiERF8* might be key regulator of ethylene production in ‘Hongyu’ mangoes. Moreover, PpERF could function as an activator in regulation of *PpPG* expression, leading to peach fruit softening (Cheng et al., 2022). It was supported by the results in this work that ETH treated fruit with lower firmness exhibited relatively higher transcription levels of *MiPG* and lower transcription levels of *MiERF2* and *MiERF8* (Figures 4G,H; Table 1). Furthermore, correlation analysis showed that the transcription of *MiPG* positively correlated with *MiERF8*, and negatively correlated with *MiERF2*, but the correlations were not significant (Figure 5). However, three EREs were found in *MiPG* promoter region (Figure 6A), which indicates that ERFs might be involved in regulating the transcription of *MiPG* through binding to the EREs.

To verify the above hypothesis, the Y1H assay was further carried out. As shown in Figure 6C, co-transformed yeast train with pHIS2-*MiPG* promoter and pGADT7-*MiERF2* or pGADT7-*MiERF8* grew well on SD/-Trp/-His/-Leu/3-AT, which suggested both MiERF2 and MiERF8 might act as mediators of fruit softening by binding to *MiPG* promoter directly. However, the special regulatory network of fruit softening mediated by MiERF2 or MiERF8 still needs further study.

In this study, we investigate the effects of ETH and 1-MCP treatment on behaviors of ethylene synthesis and postharvest softening in ‘Hongyu’ mangoes during storage at 15°C. Compared with control, ETH treatment stimulated the production of ethylene, respiratory rate and MDA accumulation, enhanced activities of PG, β-GAL and CX and expressions of *MiPG*, *Miβ-GAL* and *MiPE*, and then exhibited the accelerating effects on mango fruit ripening and softening. On the contrary, 1-MCP treatment showed the inhibitory effects, which mainly attributed to reduction of ethylene anabolism and MDA content, and lower enzymes activities and expression of genes involved in cell wall degradation. Moreover, firmness positively correlated with protopectin content and negatively correlated with soluble pectin content, which was supported by the results that mango fruit with higher firmness showed significantly lower activities of cell wall modifying enzymes and lower expressions of *MiPG* and *Miβ-GAL*. Correlation analysis further revealed that fruit softening was positively related with ethylene anabolism and *MiERF2* expression. In addition, Y1H revealed that either MiERF2 or MiERF8 might play important role in regulation of fruit softening by binding to *MiPG* promoter. These results provide more theoretical basis for postharvest ethylene control and give new perspectives about the correlation between mango fruit ripening and softening.

## Data availability statement

The original contributions presented in the study are included in the article/Supplementary material, further inquiries can be directed to the corresponding authors.

## Author contributions

JM and WL methodology. JM and WJ software, formal analysis, investigation, and data curation. HG and YS resources. RL, JM, and WL writing-original draft. RL and WL writing-review, and editing. YS and WL supervision. YS and WL project administration. RL and WL funding acquisition. All authors contributed to the article and approved the submitted version.

## Funding

This research was funded by the National Natural Science Foundation of China (grant no. 32072275), the Construction Project of Hainan Academician Team Innovation Center (grant no. HD-YSZX-202112) and the Start-Up Grant Program in Hainan University (grant no. KYQD(ZR)-22127).

## Conflict of interest

The authors declare that the research was conducted in the absence of any commercial or financial relationships that could be construed as a potential conflict of interest.

## Publisher’s note

All claims expressed in this article are solely those of the authors and do not necessarily represent those of their affiliated organizations, or those of the publisher, the editors and the reviewers. Any product that may be evaluated in this article, or claim that may be made by its manufacturer, is not guaranteed or endorsed by the publisher.

## Footnotes

^1^ https://primerexplorer.jp/e/

^2^ http://gpminer.mbc.nctu.edu.tw/

## Abbreviations

ETH: Ethephon
1-MCP: 1-Methylcyclopropene
PG: polygalacturonase
β-GAL: β-galactosidase
PE: pectin methylesterase
CX: cellulase
ERF: ethylene responsive factor
MDA: Malondialdehyde
ACS: 1-aminocyclopropane-1-carboxylate synthase
ACO: 1-aminocyclopropane-1-carboxylate oxidase
Y1H: 1yeast one hybrid
