# Supplementary material for: 1-Methylcyclopropene counteracts ethylene promotion of fruit softening and roles of MiERF2/8 and MiPG in postharvest mangoes
Source: Front Plant Sci. 2022 Sep 20;13:971050. doi: 10.3389/fpls.2022.971050 (PMC9531572; doi:10.3389/fpls.2022.971050)
Supplement: Supplementary file 1 [file Table_2.DOCX]

**Table S1 Primers used in this study.**

|  | Accession number | Gene name | [Primer](file:///D:\2022年-上半年工作\写文章-2022-初\马-软化文章\5稿%20-4-1%20全.docx#/javascript:;) [sequence](file:///D:\2022年-上半年工作\写文章-2022-初\马-软化文章\5稿%20-4-1%20全.docx#/javascript:;) (5'-3') |
| --- | --- | --- | --- |
| qRT-PCR | [XM_044641614.1](https://www.ncbi.nlm.nih.gov/entrez/viewer.fcgi?db=nucleotide&id=2118933004) | *MiPG* | F-AAGGAGGAGGGACAATCAATGG  R-GTAGGGAAGTCACAACCCGAAG |
|  | XM_044654615.1 | *MiPE* | F-ATTTACAGGTTATCCAGGTC  R-CTTCTTCTAAGCCGTTCT |
|  | XM_044628068.1 | *Miβ-GAL* | F-TGGAACGGTTTCTATGGC  R-TCCTCAACTGGTCGGTGT |
|  | XM_044619176.1 | *MiCX* | F-GTTGTGAGCCCATTTTTTTGCC  R-GCTTGGTGTTGCCTCTTTCTTT |
|  | XM_044636718.1 | *MiACS6* | F- GATTTAATCATGGACTGGATTCGCA  R- ATTTGCTCGTTTGCTCCC |
|  | XM_044609568.1 | *MiACO1* | F-CCAATGGCAAATACAAGA  R-ATGGTGGTGCTGGATAGA |
|  | XM_044622999.1 | *MiERF2* | F-AAAAGCTGAGCCGAGGGAAGAG  R-GAACCAACCCGGTGAGGAAAAT |
|  | XM_044640582.1  XM_044653145.1 | *MiERF8*  *MiACTIN* | F-TTTCTCTGTCCATCACTCGCTT  R-CATCTTTTCTTTTCGTGTTCCT  F- TTGTTAATAACTGGGATGAC  R- GAAAGAACAGCCTGAATAG |
| Y1H | XM_044622999.1  XM_044640582.1 | *MiERF2*  *MiERF8*  *MiPG-pro* | F-GAATTCATGTTCTTCAGCAGTCTGAG  R-GGATCCTTAGTCGATTCCTTGTGCAA  F-TTTCTCTGTCCATCACTCGCTT  R-CATCTTTTCTTTTCGTGTTCCT  F-CCCGGGTTGGTCTGATTGTACGATGCT  R-GAGCTCGCTCTGTTAAACAAGTATGAAC |
